# Anticancer activity of dietary xanthone α-mangostin against hepatocellular carcinoma by inhibition of STAT3 signaling via stabilization of SHP1

**DOI:** 10.1038/s41419-020-2227-4

**Published:** 2020-01-24

**Authors:** Hai Zhang, Yu-ping Tan, Lin Zhao, Lun Wang, Nai-jie Fu, Song-ping Zheng, Xiao-fei Shen

**Affiliations:** 1 Chengdu University of Traditional Chinese Medicine, Chengdu, China; 2 Key Laboratory of Birth Defects and Related Diseases of Women and Children (Ministry of Education), West China Second University Hospital, Sichuan University, Chengdu, China; 3 Chengdu Institute of Biology, Chinese Academy of Sciences, Chengdu, China; 4 Department of Neurosurgery, West China Hospital, Sichuan University, Chengdu, China

**Keywords:** Drug development, Pharmacodynamics

## Abstract

Hepatocellular carcinoma (HCC) is one of the most lethal human cancers worldwide. The dietary xanthone α-mangostin (α-MGT) exhibits potent anti-tumor effects in vitro and in vivo. However, the anti-HCC effects of α-MGT and their underlying mechanisms are still vague. Aberrant activation of signal transducer and activator of transcription 3 (STAT3) is involved in the progression of HCC. We therefore investigated whether α-MGT inhibited the activation of STAT3 and thereby exhibits its anti-HCC effects. In this study, we found that α-MGT significantly suppressed cell proliferation, induced cell cycle arrest, and triggered apoptosis in HCC cells, including HepG2, SK-Hep-1, Huh7, and SMMC-7721 cells in vitro, as well as inhibiting tumor growth in nude mice bearing HepG2 or SK-Hep-1 xenografts. Furthermore, α-MGT potently inhibited the constitutive and inducible activation of STAT3 in HCC cells. In addition, α-MGT also suppressed IL-6-induced dimerization and nuclear translocation of STAT3, which led to inhibition of the expression of STAT3-regulated genes at both mRNA and protein levels. Mechanistically, α-MGT exhibited effective inhibition of the activation of STAT3’s upstream kinases, including JAK2, Src, ERK, and Akt. Importantly, α-MGT increased the protein level of Src homology region 2 domain-containing phosphatase-1 (SHP1), which is a key negative regulator of the STAT3 signaling pathway. Furthermore, α-MGT enhanced the stabilization of SHP1 by inhibiting its degradation mediated by the ubiquitin–proteasome pathway. Knockdown of SHP1 using siRNA obviously prevented the α-MGT-mediated inhibition of the activation of STAT3 and proliferation of HCC cells. In summary, α-MGT exhibited a potent anti-HCC effect by blocking the STAT3 signaling pathway via the suppression of the degradation of SHP1 induced by the ubiquitin–proteasome pathway. These findings also suggested the potential of dietary derived α-MGT in HCC therapy.

## Introduction

Hepatocellular carcinoma (HCC) is one of the commonest human malignancies and has one of the highest mortality rates of all cancers worldwide, especially in China^1,2^. Although therapies, such as surgery, chemotherapy, and immunotherapy, have been used in the clinical treatment of HCC, the prognosis of HCC patients remains unfavorable^3^. For example, only patients with early-stage HCC are eligible for hepatectomy or liver transplantation^3^. Targeted therapies, such as sorafenib, only offer limited clinical efficacy and lead to severe adverse effects^4,5^. Therefore, there is an urgent need to find an effective adjuvant therapy to increase the survival rate of HCC patients.

Signal transducer and activator of transcription 3 (STAT3) is considered as an oncogene, because its activation plays a key role in the transcriptional regulation of genes that are involved in cell proliferation, survival, metastasis, and immune evasion^6^. A great many of clinical data also demonstrate that overexpressed and/or constitutively activated STAT3 is frequently observed in tumor cells as well as tissue samples, and contributes to tumorigenesis and progression in the majority of cancers, including HCC^6,7^. Furthermore, in comparison with cancer cells, non-cancerous cells are not sensitive to loss of STAT3 function^8^. Therefore, STAT3 is deemed to be a promising target for cancer therapy with a high therapeutic index^9^.

**Fig. 1:**
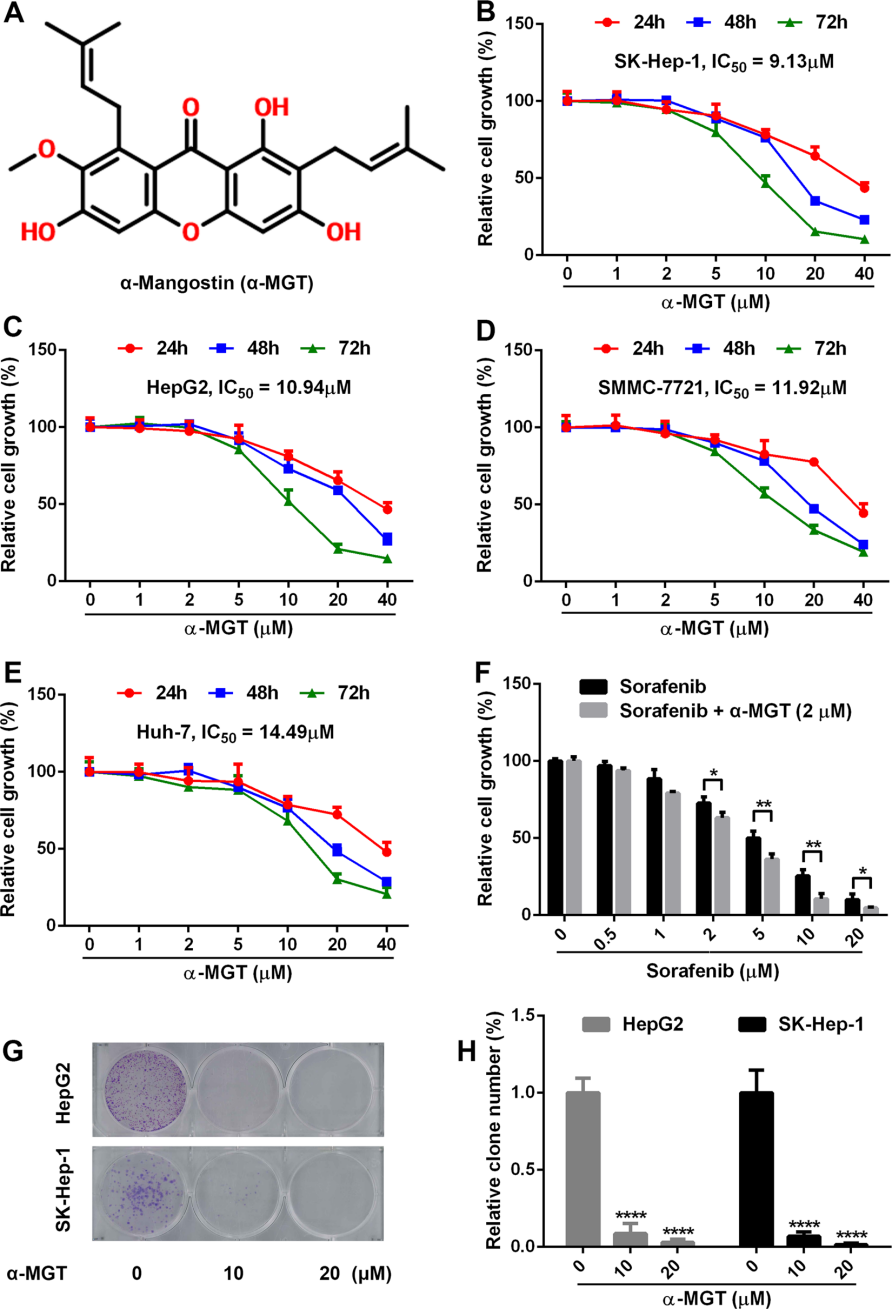
α-MGT exhibits potent growth inhibition in HCC cells in vitro. **a** Chemical structure of α-MGT. **b–e** Human HCC cells (5000 cells/well) were treated with a series of concentrations of α-MGT for the indicated times, respectively. The cell growth was then detected by SRB staining. **f** SK-Hep-1 cells were treated with the indicated concentrations of sorafenib for 48 h without or with α-MGT (2 μM). HepG2 and SK-Hep-1 cells (1000 cells/well) were treated with α-MGT for 24 h, and then incubated for additional 14 days. The numbers of clones were measured by crystal violet staining. Representative images (**g**) and quantitative results (**h**) of colony formation were presented. Data are expressed as mean ± SD, *n* = 3. *p < 0.05, **p < 0.01, ***p < 0.001, and ****p < 0.0001 vs. vehicle control.

Recently, dietary phytochemicals and natural products received considerable interest in the development of anti-tumor agents^10,11^. The compound α-mangostin (α-MGT, Fig. 1a), a naturally occurring xanthone, is the most abundant active constituent isolated from the pericarps of mangosteens^12^. Thus far, α-MGT has been proved to possess a variety of pharmacological activities, including antioxidant, anti-infective, anti-diabetic, cardioprotective, and neuroprotective properties^12,13^. Furthermore, a great deal of evidence has reported that α-MGT exerts potent anti-anticarcinogenic activity against various types of cancer cells, such as gastric cancer, colorectal cancer, and breast cancer cells^14^. In addition, α-MGT can also inhibit angiogenic and metastatic processes of tumor cells^15,16^. Thus, α-MGT is a promising lead compound to be used in cancer chemotherapy^14^. Previously, α-MGT was found to induce apoptosis in SK-Hep-1 HCC cells via the inhibition of p38 mitogen-activated protein kinase (MAPK) signaling^17^. However, the anti-HCC effect of α-MGT and its underlying molecular mechanisms are not fully understood. In this study, we found that α-MGT significantly suppresses STAT3 signaling by inhibiting degradation of Src homology region 2 domain-containing phosphatase-1 (SHP1) protein, a negative regulator of STAT3 signaling, and thereby exerts a potent anti-HCC effect.

## Results

### α-MGT inhibits the growth of HCC cells in vitro

A sulforhodamine B (SRB) staining assay was performed to evaluate the anti-proliferative activity of α-MGT against human HCC cells, including HepG2, SK-Hep-1, SMMC-7721, and Huh-7 cells. As shown in Fig. 1b–e, α-MGT inhibited the growth of HCC cells in vitro in a dose- and time-dependent manner. The half-inhibitory concentrations (IC_50_) of α-MGT in the cases of SK-Hep-1, HepG2, SMMC-7721, and Huh-7 HCC cells after treatment for 72 h were 9.44, 10.94, 13.22, and 14.49 μM, respectively. Sorafenib, a first-line drug for HCC therapy^4,5^, also significantly inhibited the cell growth of SK-Hep-1 cells with an IC_50_ value of 3.63 μM after treatment for 72 h (Fig. 1f). Moreover, α-MGT at a dose of 2 μM significantly boosted the growth inhibition of sorafenib on SK-Hep-1 HCC cells (Fig. 1f). To further assess the growth inhibition of α-MGT on HCC cells, we performed a colony formation assay in vitro. Treatment with α-MGT obviously reduced the clonogenic capacities of HepG2 and SK-Hep-1 HCC cells (Fig. 1g, h). Taken together, these results suggest that α-MGT exhibits a potent anti-proliferative effect on HCC cells in vitro.

### α-MGT induces cell cycle arrest and apoptosis in HCC cells

**Fig. 2:**
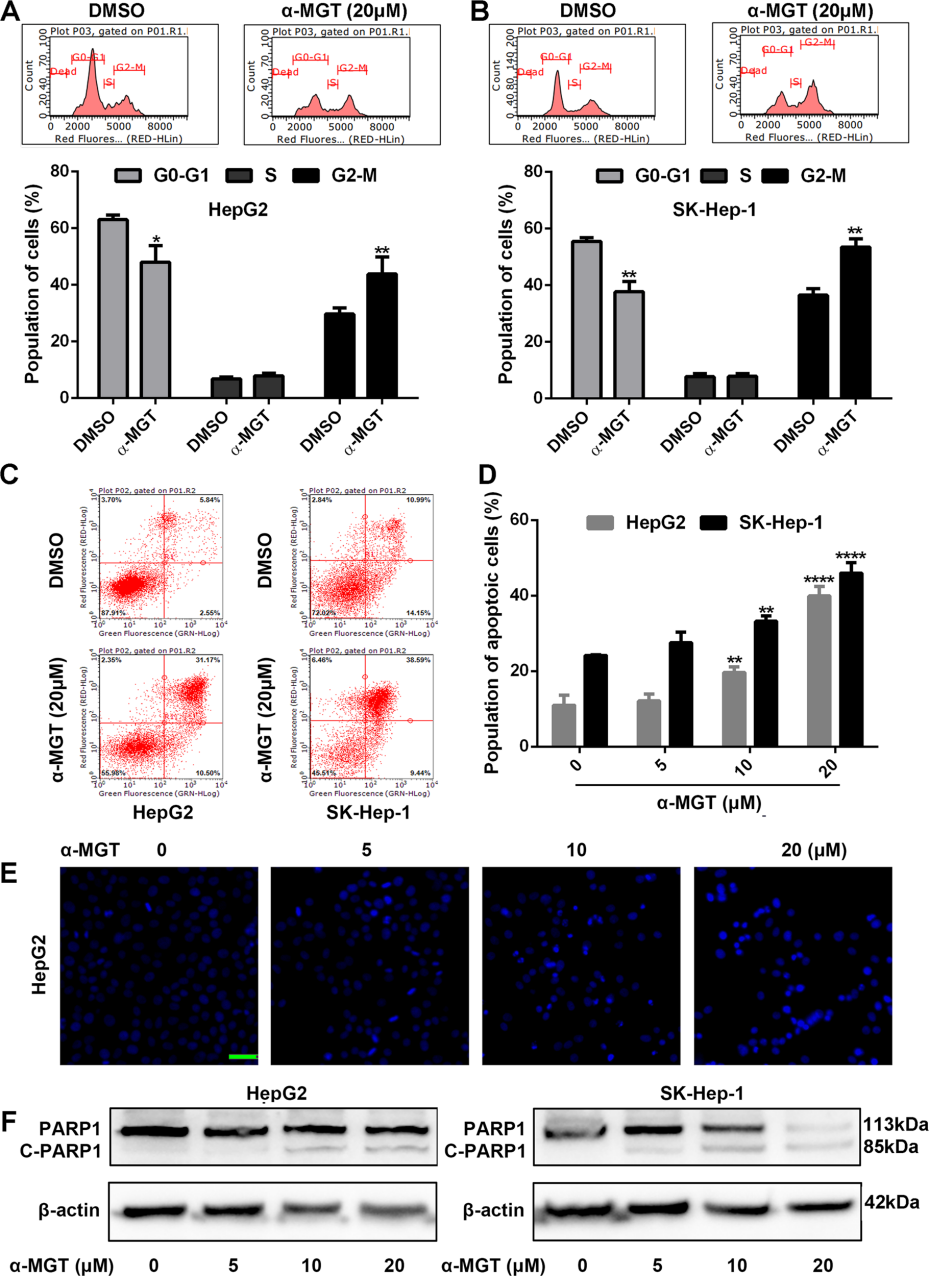
α-MGT induces cell cycle arrest and apoptosis in HCC cells in vitro. **a**, **b** HepG2 and SK-Hep-1 cells were treated with α-MGT (20 μM) for 24 h. Cell cycle distribution of HCC cells were then analyzed by flow cytometry through PI staining. **c**, **d** HepG2 and SK-Hep-1 cells were treated with DMSO- or α-MGT for 24 h, and then stained with Annexin V-FITC/PI. The population of apoptotic cells was measured by flow cytometry. **e** DMSO- or α-MGT-treated HepG2 cells were stained with DAPI, and observed by immunofluorometric microscope. Bar = 50 μm. **f** After treatment of α-MGT for 24 h, the expression of PARP and cleaved PARP were detected using western blotting assay. Data are expressed as mean ± SD, *n* = 3. *p < 0.05, **p < 0.01, ***p < 0.001, and ****p < 0.0001 vs. vehicle control.

Because regulated cell-cycle progression is essential for cell growth^18^, we thus examined the effect of α-MGT on the cell cycle of HCC cells. As shown in Fig. 2a, b, treatment with α-MGT-induced G2-M phase arrest in HepG2 and SK-Hep-1 cells in a concentration-dependent manner, respectively. Furthermore, a pro-apoptotic effect is also involved in inhibition of the growth of cancer cells, including HCC cells^11,19^. Hence, we further measured the pro-apoptotic role of α-MGT on HCC cells. An annexin V-fluorescein isothiocyanate (FITC)/propidium iodide (PI) assay showed that treatment with α-MGT caused a significant increase in the proportion of apoptotic cells among HepG2 and SK-Hep-1 cells (Fig. 2c, d). Moreover, obvious apoptotic phenotypes, such as chromatin condensation, nuclear fluorescence, and nuclear fragmentation^11,19^, were observed in α-MGT-treated HepG2 cells (Fig. 2e). Finally, western blotting showed that treatment with α-MGT for 24 h caused a notable induction of cleaved-poly ADP-ribose polymerase (PARP), a well-known biomarker of apoptosis^20^, in HepG2 and SK-Hep-1 HCC cells (Fig. 2f). Hence, α-MGT can induce cell cycle arrest and apoptosis in HCC cells in vitro.

### α-MGT inhibits the constitutive and inducible activation of STAT3 in HCC cells

**Fig. 3:**
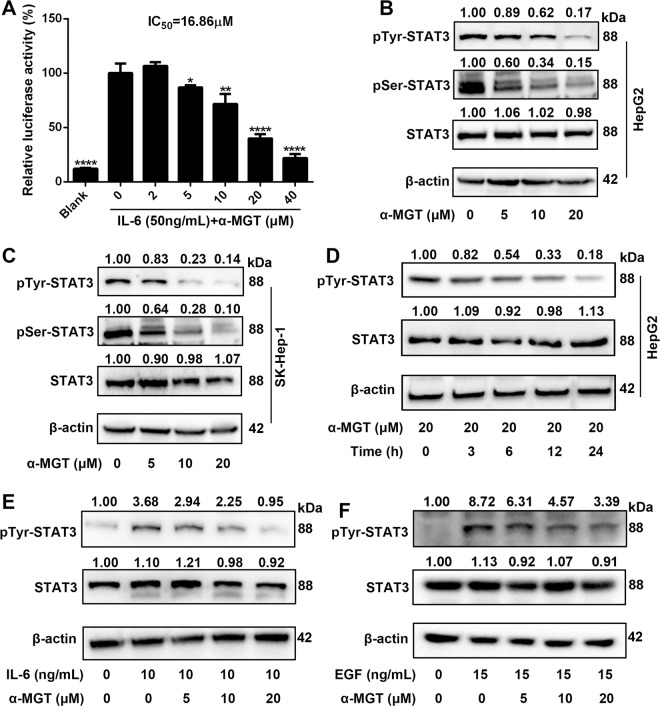
α-MGT blocks constitutive and inducible activation STAT3 in HCC cells. **a** HepG2/STAT3-luceferase reporter cells were pre-treated with α-MGT at indicated concentrations for 6 h, and luciferase activity was measured following stimulation with IL-6 (50 ng/mL) for 5 h. **b**–**d** After treatment of α-MGT for the indicated times, the phosphorylation of STAT3 were assessed using western blotting. **e**, **f** HepG2 cells were pre-treated with α-MGT for 6 h, and then incubated with IL-6 (10 ng/mL) or EGF (15 ng/mL) for additional 30 min. The phosphorylation level of STAT3 was assessed by western blotting. β-actin was used as the control. Data are expressed as mean ± SD, *n* = 3. *p < 0.05, **p < 0.01, ***p < 0.001, and ****p < 0.0001 vs. vehicle control.

STAT3 signaling is essential for the proliferation and survival of HCC cells^7^. We therefore assessed the effect of α-MGT on the activation of STAT3 using a luciferase reporter gene assay. As shown in Fig. 3a, α-MGT inhibited the interleukin-6 (IL-6)-induced activity of STAT3-responsive luciferase in HepG2 cells in a dose-dependent manner, with an IC_50_ value of 16.86 μM. To further determine whether α-MGT suppresses STAT3 activation, we evaluated the phosphorylation of STAT3 in HCC cells using western blotting analysis. We found that treatment with α-MGT suppressed the phosphorylation of STAT3 at Tyr705 and Ser727 in HepG2 cells (Fig. 3b) and SK-Hep-1 cells (Fig. 3c) in a dose-dependent fashion, respectively. Furthermore, treatment with α-MGT resulted in a time-dependent inhibition of the activation of STAT3 in HepG2 HCC cells (Fig. 3d). In addition, IL-6- or epidermal growth factor (EGF)-induced activation of STAT3 was also markedly repressed in HepG2 cells by treatment with α-MGT (Fig. 3e, f). These results demonstrate that α-MGT is a potent inhibitor of the STAT3 signaling pathway in HCC cells.

### α-MGT suppresses the dimerization and nuclear translocation of STAT3, as well as STAT3-targeted genes in HCC cells

**Fig. 4:**
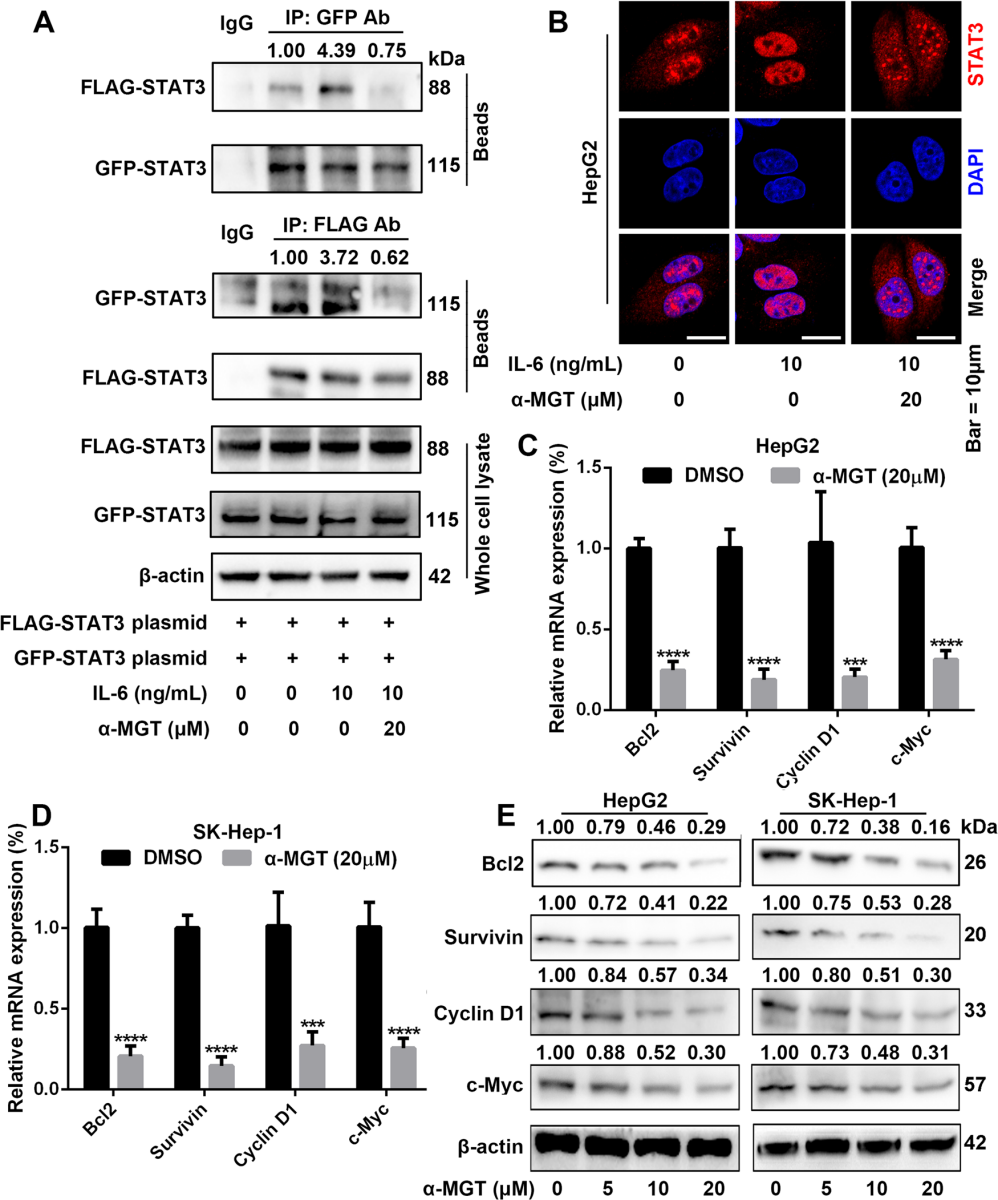
α-MGT inhibits dimerization and nuclear translocation of STAT3 and expression of STAT3 target genes in HCC cells. **a** HepG2 cells transfected with Flag- and GFP-tagged STAT3 plasmids for 24 h. Next, cells were treated with α-MGT for 6 h, followed by incubation with IL-6 (10 ng/mL) for 30 min, then subjected to immunoprecipitation, and immunoblotted with Flag or GFP antibody (Ab). Whole cell extracts were processed for western blotting with the indicated antibodies. **b** HepG2 cells were pre-treated with α-MGT for 6 h, followed by stimulating with IL-6 (10 ng/mL) for 30 min. Anti-STAT3 antibody (red) was used to locate STAT3 in cells. Cell nuclei were stained with DAPI. Bar = 10 μm. **c**, **d** HepG2 and SK-Hep-1 cells were treated with α-MGT for 24 h. The mRNA levels of Bcl2, survivin, cyclin D1, and c-Myc were measured by RT-qPCR. β-actin was used as an internal control. **e** After treatment of α-MGT for 24 h, the protein expression of Bcl2, survivin, cyclin D1, and c-Myc were analyzed using western blotting. Data are expressed as mean ± SD, n = 3. *p < 0.05, **p < 0.01, ***p < 0.001, and ****p < 0.0001 vs. vehicle control.

Phosphorylation at Tyr705 in turn drives the dimerization of STAT3 through interaction with its SH2 domain^21^. We therefore transiently co-transfected green fluorescent protein (GFP)-tagged STAT3 and FLAG-tagged STAT3 plasmids into HepG2 cells, and determined the interactions of GFP-STAT3 and FLAG-STAT3 by an immunoprecipitation (IP) assay. As shown in Fig. 4a, α-MGT at dose of 20 μM markedly decreased IL-6-induced interactions of GFP-STAT3 and FLAG-STAT3 monomers. Dimerizated STAT3 is then translocated to the nucleus, where it binds the specific DNA-response elements in the promoters of target genes to mediate transcription^21^. The results of immunofluorescence assay indicated that α-MGT effectively blocked IL-6-induced nuclear translocation of STAT3 in HepG2 cells (Fig. 4b). Furthermore, a real-time quantitative polymerase chain reaction (RT-qPCR) assay showed that α-MGT also caused marked inhibitory effects on the mRNA expression of STAT3-targetd genes in HepG2 and SK-Hep-1 cells, including B-cell lymphoma/leukemia 2 (Bcl2), survivin, cyclin D1, and v-myc myelocytomatosis viral oncogene homolog (c-Myc) (Fig. 4c, d). A western blotting assay further demonstrated that α-MGT exhibited potent suppression on the protein expression of the aforementioned STAT3-targeted genes in HepG2 cells and SK-Hep-1 cells in a concentration-dependent manner (Fig. 4e). Taken together, these results suggest that α-MGT obviously suppresses the dimerization and nuclear translocation of STAT3, as well as STAT3-targeted genes in HCC cells.

### α-MGT attenuates the phosphorylation of upstream kinases in HCC cells

**Fig. 5:**
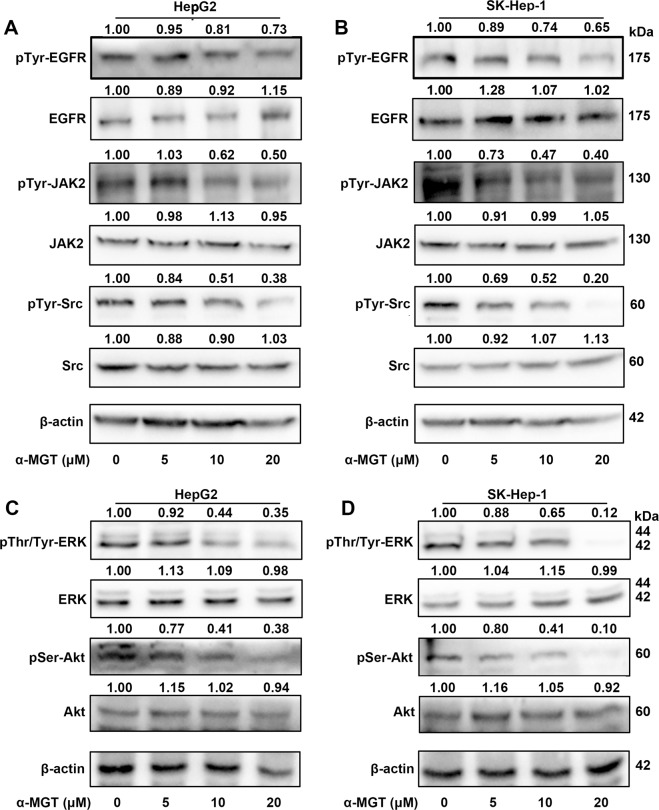
α-MGT represses the activation of STAT3’s upstream kinases in HCC cells. HepG2 and SK-Hep-1 cells were treated with the indicated concentrations of α-MGT for 24 h. Whole cell extracts were used to determine the tyrosine phosphorylation levels of EGFR, JAK2, and Src using specific antibodies (**a**, **b**). **c**, **d** The phosphorylation levels of ERK and Akt were also assessed by western blotting. β-actin was used as the control.

Several upstream tyrosine kinases, including epidermal growth factor receptor (EGFR), Janus kinase 2 (JAK2), and proto-oncogene tyrosine-protein kinase Src are responsible for the activation of STAT3^21,22^. We therefore assessed whether α-MGT alters the activation state of these kinases. As shown in Fig. 5a, b, α-MGT remarkably suppressed the tyrosine phosphorylation of JAK2 and Src in HepG2 cells and SK-Hep-1 cells in a dose-dependent manner. However, treatment with α-MGT only caused a moderate inhibition on the tyrosine phosphorylation of EGFR in HepG2 cells (Fig. 5a) and SK-Hep-1 cells (Fig. 5b). The inhibition of α-MGT on the activation of these upstream tyrosine kinases may contribute to its suppression of the phosphorylation of Tyr705 in STAT3 in HCC cells. Furthermore, serine/threonine kinases, such as extracellular signal-regulated kinase (ERK) and RAC serine/threonine protein kinase (Akt), are involved in the phosphorylation of STAT3 at Ser727^23,24^. Our results showed that α-MGT also dose-dependently attenuated the activation of ERK and Akt in HepG2 cells and SK-Hep-1 cells (Fig. 5c, d), which may contribute to its inhibition of Ser727-phosphorylated STAT3. Altogether, α-MGT effectively blocks the phosphorylation of upstream kinases in HCC cells.

### Tyrosine phosphatase SHP1 is involved in α-MGT-mediated action in HCC cells

**Fig. 6:**
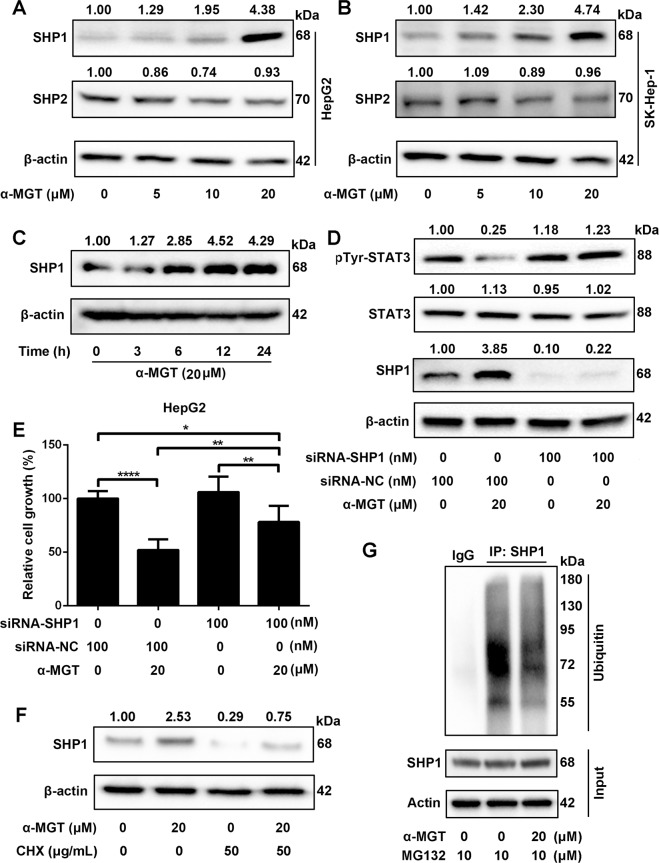
SHP1 is important for α-MGT-mediated actions in HCC cells. **a**, **b** The expression of SHP1 and SHP2 were detected in α-MGT- or vehicle-treated HepG2 or SK-Hep-1 cells by western blotting assay. **c** HepG2 cells were treated with α-MGT for the indicated times. The expression of SHP1 was measured using western blotting. β-actin was used as the loading control. HepG2 cells were transfected with SHP-1 siRNA or scrambled siRNA. After 24 h, cells were treated with α-MGT (20 μM) for additional 24 h. Next, cells were lysed and applied to immunoblotting with the indicated antibodies (**d**), or measured by SRB staining (**e**). **f** HepG2 cells were pre-treated with α-MGT (20 μM) for 6 h, and then exposed to CHX (50 μg/mL) for additional 9 h. The protein level of SHP1 was detected by western blotting. **g** HepG2 cells were pre-treated with MG132 (10 μM) for 6 h, and followed by incubation with α-MGT (20 μM) for 9 h. The cell lysates were then subjected to immunoprecipitation using IgG or SHP1 antibody, and immunoblotted with the indicated antibodies. Data are expressed as mean ± SD, *n* = 3. *p < 0.05, **p < 0.01, ***p < 0.001, and ****p < 0.0001 vs. control.

Protein tyrosine phosphatases (PTPases), including SHP1 and SHP2, have been reported to be involved in the inactivation of STAT3 signaling^25^. We further investigated whether α-MGT could block STAT3 activation via a PTPase pathway. RT-qPCR results showed that α-MGT did not change the mRNA expression of SHP1 and SHP2 in HepG2 cells and SK-Hep-1 cells (Supplementary Fig. 1). Interestingly, we found that treatment with α-MGT induced a noticeable elevation in the protein expression of SHP1 in a dose- and time-dependent manner (Fig. 6a–c), whereas no significant change in the expression of SHP2 was found after the addition of α-MGT in both HepG2 cells (Fig. 6a) and SK-Hep-1 cells (Fig. 6b). Furthermore, measurement of PTPase activity indicated that α-MGT (1–200 μM) did not change the activity of SHP1 or SHP2 in vitro (Supplementary Fig. 2). We therefore speculated that an elevated protein level of SHP1 might be involved in α-MGT-mediated inactivation of STAT3. To confirm this hypothesis, we knocked down the expression of SHP1 in HepG2 cells using small interfering RNA (siRNA). As shown in Fig. 6d, α-MGT-induced expression of SHP1 was effectively decreased in the cells transfected with SHP1 siRNA, but not in those transfected with the negative control (NC) siRNA. Furthermore, we also found that α-MGT failed to inhibit the activation of STAT3 in the cells treated with SHP1 siRNA, but still effectively blocked STAT3 activation in the cells treated with the NC siRNA (Fig. 6d). In addition, the SRB assay showed that α-MGT-induced growth inhibition on HepG2 cells could be mostly prevented by treatment of SHP1 siRNA, but not by the NC siRNA (Fig. 6e). Hence, our results suggest that SHP1 plays a pivotal role in α-MGT-mediated actions in HCC cells.

The quantity of protein is precisely controlled by the ubiquitin–proteasome pathway^26^. This prompted us to ask whether α-MGT-induced increase in protein level of SHP1 can be attributed to an inhibitory role of α-MGT on the ubiquitin–proteasome pathway. To demonstrate the inhibitory effect of α-MGT on the degradation of SHP1, we used cycloheximide (CHX) to inhibit protein synthesis. As shown in Fig. 6f, after exposure to CHX for 9 h, the protein level of SHP1 was markedly decreased in HepG2 cells, whereas α-MGT significantly rescued the reduction in the protein level of SHP1, which suggests that α-MGT enhances the stability of SHP1 protein. Furthermore, in the presence of the proteasome inhibitor MG132, α-MGT significantly suppressed the ubiquitination of SHP1 in HepG2 cells, as measured by an IP assay (Fig. 6g). These data indicate that the inhibitory effect of α-MGT on the activation of STAT3 involves stabilizing SHP1 protein by suppressing the ubiquitin–proteasome pathway.

### α-MGT represses the growth of human HCC xenograft tumor in vivo

**Fig. 7:**
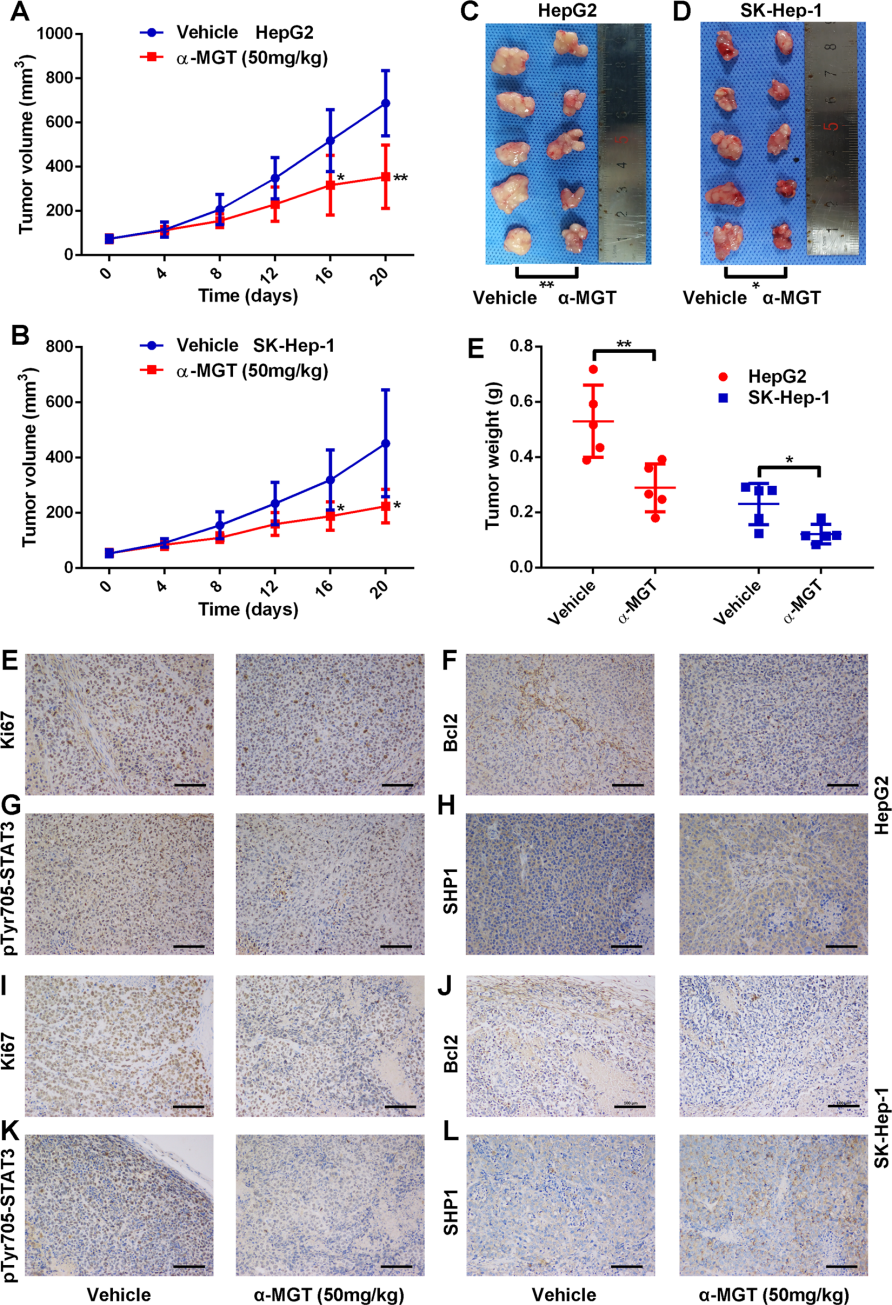
α-MGT suppresses HepG2 and SK-Hep-1 xenograft tumor growth in nude mice. The nude mice bearing HepG2 or SK-Hep-1 cells were intraperitoneally administered vehicle or α-MGT (50 mg/kg) for 20 days. The tumor volumes (**a**–**d**) and tumor weights (**e**) were measured. The expressions of Ki67 (**e**, **i**), Bcl2 (**f**, **j**), pTyr705-STAT3 (**g**, **k**), and SHP1 (**h**, **l**) in tumor tissues were evaluated by IHC method. Bar = 100 μm. Data are expressed as mean ± SD, *n* = 5. *p < 0.05, **p < 0.01, ***p < 0.001, and ****p < 0.0001 vs. vehicle control.

To determine whether α-MGT inhibits the growth of HCC tumors in vivo, we established xenografts of human HCC tumors in nude mice using HepG2 cells and SK-Hep-1 cells. When the tumors were palpable, the mice were intraperitoneally injected with vehicle or α-MGT (once per day) for 20 days. In comparison with the vehicle control, α-MGT administered at 50 mg/kg significantly inhibited the growth of HCC tumors in terms of tumor volume (Fig. 7a–c) and tumor weight (Fig. 7d). Furthermore, in comparison with the vehicle-treated mice, no significant loss of body weight was observed in nude mice treated with α-MGT (Supplementary Fig. 3A, B). In addition, treatment with α-MGT did not cause pathological changes in the heart, liver, spleen, lung, or kidney (Supplementary Fig. 3C, 3D). Moreover, the expression of the proliferation marker antigen identified by monoclonal antibody Ki-67 (Ki67) (Fig. 7e, i) and the anti-apoptotic protein Bcl2 (Fig. 7f, j) was repressed by α-MGT, in comparison with the vehicle. Additionally, we also found that α-MGT inhibited the phosphorylation of Tyr705 in STAT3 (Fig. 7g, k) and induced the expression of SHP1 (Fig. 7h, l) in xenograft tumors in mice bearing HepG2 cells and SK-Hep-1 HCC cells. Taken together, these data indicated that α-MGT inhibits the growth of human HCC tumors in vivo.

### α-MGT inhibits the growth of other STAT3-driven tumor cells in vitro

**Fig. 8:**
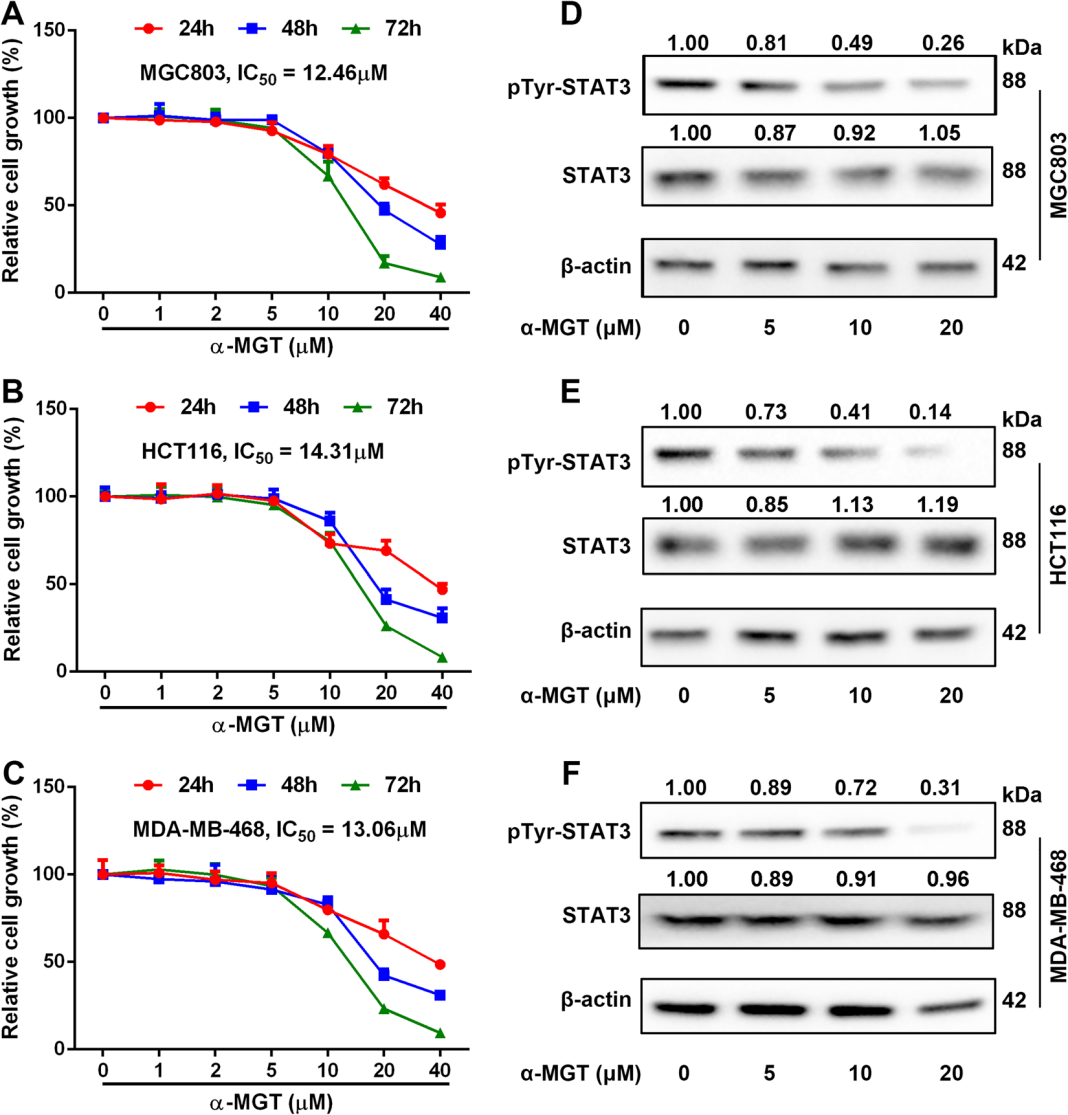
α-MGT inhibits the growth of tumor cells harboring constitutive activation of STAT3 in vitro. Human cancer cells, including MGC803 (**a**), HCT116 (**b**), and MDA-MB-468 (**c**) cells (5000 cells/well) were treated with a series of concentrations of α-MGT for the indicated times, respectively. The cell growth was then detected by SRB staining. After treatment of α-MGT for 24 h, the tyrosine phosphorylation of STAT3 was determined using western blotting in MGC803 (**d**), HCT116 (**e**), and MDA-MB-468 (**f**) cells. Data are expressed as mean ± SD, *n* = 3.

In light of the important role of STAT3 in tumor development and progression in several types of cancer, we wondered whether α-MGT could inhibit the growth of other tumor cells harboring constitutive activation of STAT3. As shown in Fig. 8a–c, treatment with α-MGT resulted in a dose- and time-dependent growth arrest of MGC803 gastric cancer cells, HCT116 colorectal cancer cells, and MDA-MB-468 breast cancer cells, respectively. The IC_50_ values of α-MGT for the growth inhibition of MGC803, HCT116, and MDA-MB-468 cell lines after treatment for 72 h were 12.46, 14.31, and 13.06 μM, respectively. Furthermore, Tyr705 phosphorylation of STAT3 was remarkably suppressed by treatment with α-MGT in these non-HCC cell lines (Fig. 8d–f).

## Discussion

The discovery of small-molecule compounds for the treatment of HCC contributes to improvements in the clinical treatment and the prognosis of patients^4,5^. In this study, we found that the natural compound α-MGT inhibited the constitutive and inducible activation of STAT3 in human HCC cells in parallel with the suppression of upstream kinases activation. Furthermore, α-MGT decreased the expression of STAT3-regulated genes, including Bcl2, survivin, cyclin D1, and c-Myc in HCC cells. We also found that α-MGT inhibited the growth of HCC cells in vitro and in vivo. Mechanistically, we demonstrated the critical role of SHP1 in α-MGT-induced anti-HCC effect. We further identified that the ubiquitin–proteasome pathway is involved in α-MGT-mediated stabilization of SHP1 protein.

STAT3 is constitutively activated in ~70% of human cancers, and is downstream of many oncogenic signals and mutations, such as a series of growth factors (e.g., EGF) and cytokines (e.g., IL-6) as well as JAK1 mutants (e.g., G1097D)^6,26^. Overactive STAT3 then promotes the expression of genes involved in proliferation, anti-apoptosis, metastasis, and inflammation, which collectively contribute to malignant transformation and tumor progression^6,7,27^. Clinically, hyperactivation of STAT3 is closely related to poor prognosis and chemotherapy resistance^28,29^. Recently, several STAT3 inhibitors such as OPB-31121 have been undergoing phase I/II clinical trials in HCC therapy^30^. Therefore, STAT3 is a very attractive target for the therapy of various cancers, including HCC^6^.

The multi-step process of STAT3 activation includes multiple points at which its oncogenic activity may be suppressed. Targeting upstream kinases is the commonest strategy used to inhibit the activation of STAT3^27^. For example, we previously found that pseudolaric acid B suppressed the phosphorylation of STAT3 by inhibiting the activation of several kinases, including EGFR, Src, ERK, and Akt^19^. Another natural compound, 1*β*-hydroxyl-5*α*-chloro-8-epi-xanthatin, also inhibited STAT3 activation via inactivating JAK2^11^. In this study, we found that α-MGT effectively inhibited the activation of tyrosine kinases, including JAK2 and Src, which are responsible for the phosphorylation of Tyr705 in STAT3. Correspondingly, a marked reduction in phosphorylation of Tyr705 in STAT3 was observed in α-MGT-treated HCC cells. The phosphorylation of STAT3 at Tyr705 is essential for its dimerization, nuclear import, and gene transcription^6,21^. As expected, we further showed that α-MGT inhibited not only the IL-6-induced association of FLAG-STAT3 and GFP-STAT3 but also the subsequent nuclear translocation of STAT3. Furthermore, the transcriptional activation of STAT3 can be regulated by phosphorylation at Ser727 via the ERK and Akt pathways^23,24^. Interestingly, the present findings also showed that α-MGT inhibited the activation of ERK and Akt in parallel with the suppression of the phosphorylation of Ser727 in STAT3 in HCC cells. Our results are partially in agreement with a previous report that α-MGT down-regulated the ERK and Akt signaling pathways in human chondrosarcoma cells. In addition, α-MGT also suppressed the activation of STAT3 induced by IL-6 or EGF, two major growth factors of tumour cells that activate STAT3^27^. In accordance with the above-mentioned results, we further found that α-MGT remarkably decreased the expression of STAT3-targeted genes, such as anti-apoptotic Bcl2 and survivin as well as proliferative cyclin D1 and c-Myc at both mRNA and protein levels in HCC cells. Considering the key role of STAT3 in HCC, the anti-HCC effects of α-MGT in vitro and in vivo can be attributed, at least in part, to its inhibition of on STAT3 signaling. In addition, hyperactivation of STAT3 is also involved in the development of several autoimmune diseases, including rheumatoid arthritis (RA) and inflammatory bowel disease (IBD). In the present study, α-MGT was observed to inhibit STAT3 signaling pathway, which thus provides further support for its therapeutic effects in RA^31^ and IBD^32^.

Although Shan et al. have found that α-MGT indeed can suppress the constitutive activation of STAT3 in human gastric adenocarcinoma cells^33^; they did not explore the detailed mechanisms involved. The activation of STAT3 signaling is precisely regulated by multiple mechanisms. In addition to the positive regulation by upstream kinases, the inactivation of JAKs, receptor tyrosine kinases, and STAT3, via dephosphorylation as a result of the interactions of PTPases (such as SHP1 and SHP2) is also very pivotal for the negative regulation of STAT3 signaling^25^. For example, SHP1 is considered to be a negative regulator of the JAK2/STAT3 pathway in HCC^34^. A marked reduction in the expression of SHP1 has been regarded as a characteristic of pathogenesis in several types of cancers, including HCC^35^. Tai et al have reported that boosting the activity of SHP1 via sorafenib or SHP1 agonists gives rise to powerful anti-HCC effects in vitro and in vivo^36^. Several natural compounds, such as emodin and evodiamine also exhibit potent anti-HCC effects by up-regulating the protein expression of SHP1^37,38^. Therefore, SHP1 is a candidate target for HCC therapy. In this study, we found that α-MGT up-regulated the protein expression of SHP1, and the suppression of SHP1 expression by siRNA mostly prevented α-MGT-mediated inactivation of STAT3 and the consequent anti-proliferative effects on HCC cells. SHP1 is also involved in the inactivation of some tyrosine kinases, including JAKs and EGFR^39,40^. Hence, α-MGT-mediated up-regulation of SHP1 protein may also contribute to its inhibition of upstream tyrosine kinases. Furthermore, α-MGT did not significantly affect the protein expression of SHP2 in HCC cells. SHP2 is responsible for the activation of the RAS/MEK/ERK and PI3K/Akt pathways^41^. We thus speculated that the inhibitory effect of α-MGT on the ERK and Akt pathways may be independent of SHP2 in HCC cells. In addition, knockdown of SHP1 did not completely prevent the α-MGT-mediated growth inhibition on HCC cells, which suggests that other mechanisms are involved in α-MGT-mediated effects.

Although previous studies have demonstrated that the some compounds can increase the protein level of SHP1, the precise mechanism of this effect has yet to be clarified. Recent studies suggested that SHP1 could be ubiquitinated and degraded into the ubiquitin–proteasome pathway in colorectal cancer and gastric cancer^42,43^. In addition, the ubiquitin–proteasome system functions as a key regulator of protein homeostasis, and plays an important role in a series of diseases, including cancers^44–46^. Interestingly, we have discovered that emodin is a 26S proteasome inhibitor^47^, which may contribute to its stabilizing effect on SHP1 protein as well as its inhibitory role on the STAT3 pathway. Therefore, targeting the ubiquitin–proteasome pathway may be a potential strategy for cancer therapy^48^. In the present study, we found that α-MGT inhibited the ubiquitination of SHP1, and thereby enhanced the stabilization of SHP1. We therefore speculated that the inhibitory effect of α-MGT on ubiquitin–proteasome pathway-mediated SHP1 degradation may be the dominant contributor to its inactivation of STAT3 signaling.

In conclusion, the present findings indicate that the anti-HCC effect of α-MGT is mediated by the suppression of the STAT3 signaling cascade via stabilizing SHP1 protein in an ubiquitin–proteasome-dependent manner (Supplementary Fig. 4). These results also suggest that α-MGT is a promising candidate for HCC therapy.

## Materials and methods

### Chemicals and reagents

The natural product α-MGT was provided by PUSH Bio-technology (Chengdu, China) and dissolved in dimethyl sulfoxide (DMSO). Cycloheximide and MG132 were purchased from Selleck (Shanghai, China). Primary antibodies against ERK, phospho-ERK (Thr202/Tyr204), Akt, phospho-Akt (Ser473), EGFR, phospho-EGFR (Tyr1125), Src, phospho-Src (Tyr527), JAK2, phospho-JAK2 (Tyr1007), STAT3, phospho-STAT3 (Tyr705), phospho-STAT3 (Ser727), Bcl2, survivin, cyclin D1, c-Myc, and ubiquitin were purchased from SAB (Nanjing, China). Primary antibodies against PARP, SHP1, SHP2, and β-actin were obtained from Proteintech (Wuhan, China) and Santa Cruz Biotechnology (Santa Cruz, USA), respectively.

### Cell culture

Human HCC cell lines, including HepG2, Huh-7, SK-Hep-1, and SMMC-7721 cells, were purchased from the Cell Bank of Chinese Academy of Sciences (Shanghai, China). HepG2 cells and SK-Hep-1 cells were cultured in minimum Eagle’s medium (MEM), whereas Huh-7 cells and SMMC-7721 cells were cultured in Dulbecco’s modified Eagle’s medium (DMEM) at 37 °C in humidified air with 5% CO_2_. Both cell culture mediums contained 10% fetal bovine serum (FBS) and 1% penicillin/streptomycin (HyClone, Logan, USA). The passage number for all HCC cells was between 4 and 15.

### SRB staining assay

The SRB assay was used to assess the anti-proliferative activity of α-MGT against HCC cells. Briefly, HCC cells (5000 cells/well) were seeded into 96-well plates, and incubated overnight. Then, the cells were treated with a series of concentrations of α-MGT for 72 h. Next, the culture medium was removed, and the cells were then fixed with 10% trichloroacetic acid, and stained with 1% SRB solution. After washing with 1% acetic acid solution, the bound SRB was dissolved with 10 mM Tris solution. The absorbance at 570 nm was detected using a microplate reader (Multiskan FC, Thermo, USA).

### Colony formation assay

Briefly, approximately 1000 HCC cells were seeded into a six-well plate and incubated overnight at 37 °C. The HCC cells were then treated with α-MGT (5 and 10 μM) for 24 h. Next, the medium containing α-MGT was removed and fresh medium was added. The HCC cells were cultured for a further 12 days, and were subsequently fixed with 4% paraformaldehyde (PFA) for 15 min, and then stained with 0.5% crystal violet for 15 min at room temperature. Finally, the numbers of cell colonies were counted.

### Cell cycle analysis

The effect of α-MGT on the cell cycle of HCC cells was assessed using PI staining. In brief, DMSO- or α-MGT-treated HCC cells were fixed with pre-chilled 70% ethanol at −20 °C overnight. After being centrifugated, the cells were incubated with 100 μg/mL of RNAse and 50 μg/mL of PI for 30 min at 37 °C in the dark. The cell-cycle distribution was then examined with a Guava EasyCyte flow cytometer (Millipore, Boston, USA).

### Apoptosis assay

Annexin V-FITC/PI double staining was used to evaluate the pro-apoptotic effect of α-MGT on HCC cells. Briefly, DMSO- or α-MGT-treated HCC cells were digested with ethylenediaminetetraacetic acid (EDTA)-free trypsin. After being centrifugated, the cells were harvested and resuspended in 500 μL of 1 × binding buffer. The cells were then stained with 5 μL of Annexin V-FITC (20 μg/mL) and PI (50 μg/ml) for 15 min at room temperature in the dark. Next, apoptotic and normal cells were detected using a Guava EasyCyte flow cytometer. Apoptosis was further examined using Hoechst 33342 staining. Briefly, DMSO- or α-MGT-treated HepG2 cells were fixed with 4% PFA for 15 min, and then stained with 5 μg/mL of Hoechst 33342 for 10 min at room temperature. Finally, the nuclear morphology was observed using a fluorescence microscopy (Olympus IX53, Tokyo, Japan).

### Luciferase activity assay

HepG2 cells were transfected with a STAT3-luciferase reporter plasmid (Promega, Madison, USA) using GenJet^TM^ DNA transfection reagent (SignaGen, Jinan, China) for 24 h. Subsequently, the transfected cells (2 × 10^5^ cells/well) were seeded into a 24-well plate and incubated overnight. Next, cells were treated with vehicle or various concentrations of α-MGT for 6 h and followed by stimulation with 50 ng/mL of IL-6 for 5 h. STAT3-luciferase activity was assessed using a luciferase kit according to the manufacturer’s instruction (Promega, Madison, USA).

### Western blotting

After being treated with α-MGT, HCC cells were lysed with pre-chilled radio-immunoprecipitation assay (RIPA) buffer containing 1% proteases and phosphatase inhibitors (Biomake, Shanghai, China). After centrifugation, the cell lysates were mixed with loading buffer, and boiled for 5 min at 98 °C. Next, the cell lysates were separated on sodium dodecyl sulfate-polyacrylamide gel (SDS-PAGE). The proteins were then blotted onto poly-(vinylidene fluoride) (PVDF) membrane (Millipore, Bedford, USA). Afterwards, the membranes were blocked with 5% non-fat milk powder in Tris-buffered saline with Tween-20 (TBST), and incubated with specific primary antibodies (1:1000) overnight at 4 °C. After being washed three times with TBST buffer, the membranes were incubated with horseradish peroxidase (HRP)-conjugated secondary antibodies (1:5000, Proteintech, Wuhan, China) for 1 h at room temperature and then washed with TBST three times. Immunoblotting signals were detected using chemiluminescent HRP substrate (Millipore, Massachusetts, USA). The optical densities of the immunoblotting bands were determined using QuantityOne® software (Bio-Rad, Hercules, USA).

### Immunoprecipitation assay

HepG2 cells were co-transfected with GFP-STAT3 and FLAG-STAT3 plasmids using GenJet^TM^ DNA transfection reagent for 24 h. Next, the cells were treated with α-MGT or DMSO for 6 h, followed by stimulating with IL-6 (10 ng/μL) for 30 min. The HepG2 cells were then collected with lysis buffer. Equal amounts of proteins were incubated with anti-GFP or anti-FLAG antibody (1 μg/mL) overnight at 4 °C. The immunoprecipitates were incubated with protein A/G agarose beads (Bimake, Shanghai, China), and non-specific proteins were washed with lysis buffer. IgG antibody (Beyotime, Shanghai, China) was used as the negative control. The immunoprecipitates were subjected to SDS-PAGE electrophoresis, and immunoblotted with anti-GFP or anti-FLAG antibody.

### Small interfering RNA transfection

HepG2 cells (2.5 × 10^5^ cells/well) were seeded into a six-well plate and allowed to adhere overnight. Afterwards, 5 μL of Lipofectamine 3000 (Invitrogen, Carlsbad, USA) was added to SHP1-targeted siRNA (50 nM sequence 1 and 50 nM sequence 2) or NC siRNA (100 nM) in 100 μL of Opti-MEM^TM^ medium (Thermo Fisher, Burlington, USA). After being transfected for 48 h, the cells were treated with α-MGT for 24 h. SHP1 siRNA sequence 1: 5′-CUG GUG GAG CAU UUC AAG ATT-3′; SHP1 siRNA sequence 2: 5′-CGC AGU ACA AGU UCA UCU ATT-3′^38^; NC siRNA sequence: 5′-AAG GAG GCT GAA CAT TCC GTC-3′ (Sangon Biotech, Shanghai, China).

### Immunofluorometric assay

HepG2 cells (approximately 1 × 10^5^) were cultured on the sterile glass coverslips in a 24-well plate overnight, and then pre-treated with α-MGT (20 μM) for 6 h prior to being stimulated with IL-6 (10 ng/mL) for 30 min. After being stimulated, the cells were washed with phosphate-buffered saline (PBS), and then fixed with 4% PFA for 15 min and permeabilized with 0.1% Triton X-100 for 15 min at room temperature. Next, the cells were blocked with 2% bovine serum albumin (BSA) and 10% goat serum for 1 h, and incubated with anti-STAT3 primary antibody (1:200) for 4 h, followed by incubation with a Cy3-labeled goat anti-rabbit IgG (1:1000, Beyotime, Shanghai, China) for 1 h at room temperature. The nuclei were stained with Hoechst 33342 (Sigma, Shanghai, China) for 5 min at room temperature in the dark. Finally, the subcellular localization of STAT3 was examined visually with a confocal microscope (FV1000, Olympus, Japan).

### Real-time quantitative polymerase chain reaction

Briefly, total RNA was then extracted using a UNIQ-10 column total RNA purification kit (Sangon, Shanghai, China). The cDNA was then preparated from the total RNAs using an All-in-One cDNA Synthesis SuperMix Kit (Bimake, Shanghai, China). Next, equal amounts of cDNAs were subjected to RT-qPCR with SYBR Green (Bimake, Shanghai, China) using a CFX96 Real-Time PCR System (Bio-Rad, Hercules, USA). The relative expression of the target genes were calculated by 2^−ΔΔCt^ method using the β-actin gene as a reference gene. The following primers were used: Bcl2 forward primer: 5′-AGT TCG GTG GGG TCA TGT GT-3′, Bcl2 reverse primer: 5′-CCA GGA GAA ATC AAA CAG AGG C-3′; survivin forward primer: 5′-CAT GTA CGT TGC TAT CCA GGC-3′, survivin reverse primer: 5′-CTC CTT AAT GTC ACG CAC GAT-3′; cyclin D1 forward primer: 5′-AAC TAC CTG GAC CGC TTC CT-3′, cyclin D1 reverse primer: 5′-CCA CTT GAG CTT GTT CAC CA-3′; c-Myc forward primer: 5′-TAC AAC ACC CGA GCA AGG AC-3′, c-Myc reverse primer: 5′-TCG TCG CAG TAG AAA TAC GG-3′; SHP1 forward primer: 5′-TGG CGT GGC AGG AGA ACA G-3′, SHP1 reverse primer: 5′-CAG TTG GTC ACA GAG TAG GGC-3′; β-actin forward primer: 5′-CAT GTA CGT TGC TAT CCA GGC-3′, β-actin reverse primer: 5′-CTC CTT AAT GTC ACG CAC GAT-3′.

### Detection of PTPase activity

The effects of α-MGT on the activity of SHP1 and SHP2 were measured using the fluorogenic 6,8-difluoro-4-methylumbelliferyl phosphate (DiFMUP, Sigma, Shanghai, China) as the substrate^49^. Briefly, purified recombinant SHP1 or SHP2 protein (20 nM) was incubated with a series of concentrations of α-MGT at 37 °C for 30 min in reaction buffer (total volume = 100 μL, 25 mM 3-morpholinopropanesulfoinc acid, pH = 7.0, 50 mM NaCl, 0.05% Tween 20, 1 mM dithiothreitol, 20 μM DiFMUP, 10 nM Microcystin LR). Next, the reaction was initiated by the addition of DiFMUP. After incubation for 30 min at 37 °C, the fluorescence signal at an excitation wavelength of 355 nm and an emission wavelength of 460 nm was determined. Sodium vanadate (Sigma, Shanghai, China) was used as the positive control.

### Tumor xenograft study

All animal experiments were carried out in accordance with the Guide for the Care and Use of Laboratory Animals published by the US National Institutes of Health (NIH publication No. 85-23, revised 1996). All animal experiments were approved by the Ethical Committee of Chengdu University of Traditional Chinese Medicine (2019-03). HepG2 or SK-Hep-1 cells (1 × 10^6^) were subcutaneously injected into the right flanks of male BALB/c nude mice (6-weeks-old, 18–20 g, Jiangsu GemPharmatech, Nanjing, China). α-MGT was dissolved into the mixed solution containing absolute alcohol, Tween-80, and sterile normal saline (1:1:8, v/v/v). Once the volumes of the resulting tumors reached ~50 mm^3^, the mice were randomly divided into two groups based on the body weight and tumor volume. The mice were then intraperitoneally injected with α-MGT (50 mg/kg) or vehicle for 20 days (once a day, 5 mice/each group). The tumor volumes and body weights were measured once every 4 days. The tumor volume (TV) was measured as TV = (0.5 × tumor length × tumor width^2^). Finally, the mice were sacrificed, and the tumors were then removed and weighed. The tumors were fixed in 4% PFA and then embedded in paraffin for immunohistochemistry (IHC) assay. Furthermore, several important organs, including the heart, liver, spleen, lung, and kidney, were also fixed in 4% PFA and embedded in paraffin. The sections were then subjected to hematoxylin and eosin staining.

### Immunohistochemistry assay

The paraffin sections were dewaxed with xylene, and then hydrated with an ethanol gradient. The sections were then treated in a pressure cooker for 10 min in 0.01 M sodium citrate buffer (pH = 6.0), and blocked with 10% goat serum and 2% bovine serum albumin (BSA) at room temperature for 1 h. Next, the sections were stained with specific antibodies against Ki67 (1:200, SAB), Bcl2 (1:100, SAB), pTyr705-STAT3 (1:100, SAB), and SHP1 (1:200, Proteintech) at 4 °C overnight. After being washed with TBST three times, the sections were incubated with 3% hydrogen peroxide for 15 min to inactivate endogenous peroxidase. Next, the sections were incubated with a HRP-labeled secondary antibody (1:1000, Proteintech) for 1 h at room temperature. Next, a diaminobenzidine (DAB) kit (Zhongshan Golden Bridge Biotechnology, Beijing, China) was used to visualize the antibody-binding sites. Thereafter, the sections were stained with hematein for 10 min, dehydrated with an ethanol gradient, cleared in xylene, and then sealed with neutral balsam on slides. Finally, the sections were observed under light microscope.

### Statistical analysis

All experiments were repeated at least three times and representative results were presented. The data are expressed as the mean ± standard deviation (SD), and were compared by one-way ANOVA followed by a Tukey’s test using GraphPad Prism 5.0 software (La Jolla, California, USA). Differences were considered statistically significant when *p* < 0.05.

## Supplementary information

Supplementary Figure 1

Supplementary Figure 2

Supplementary Figure 3

Supplementary Figure 4
