# Supplementary figures and images for: Anticancer activity of dietary xanthone α-mangostin against hepatocellular carcinoma by inhibition of STAT3 signaling via stabilization of SHP1
Source: Cell Death Dis. 2020 Jan 24;11(1):63. doi: 10.1038/s41419-020-2227-4 (PMC6981176; doi:10.1038/s41419-020-2227-4)

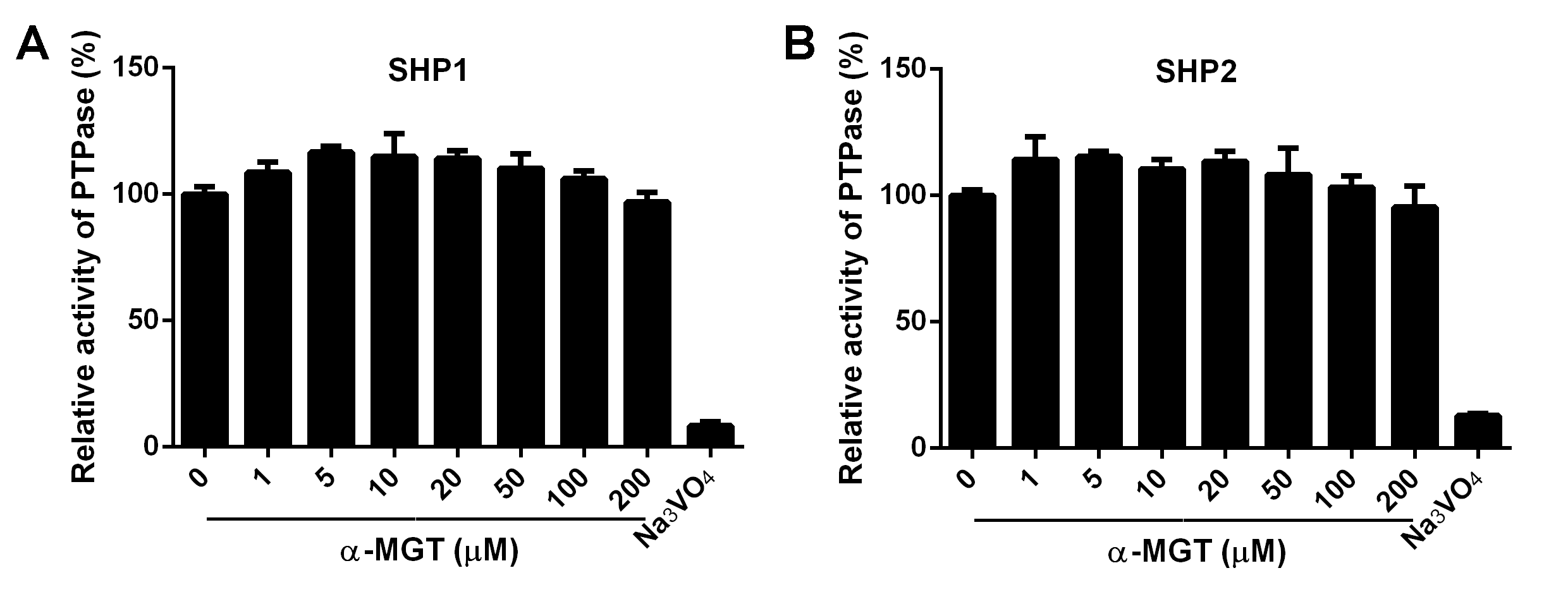

Supplement: Supplementary file 2 — Supplementary Figure 2 [file 41419_2020_2227_MOESM2_ESM.tif]

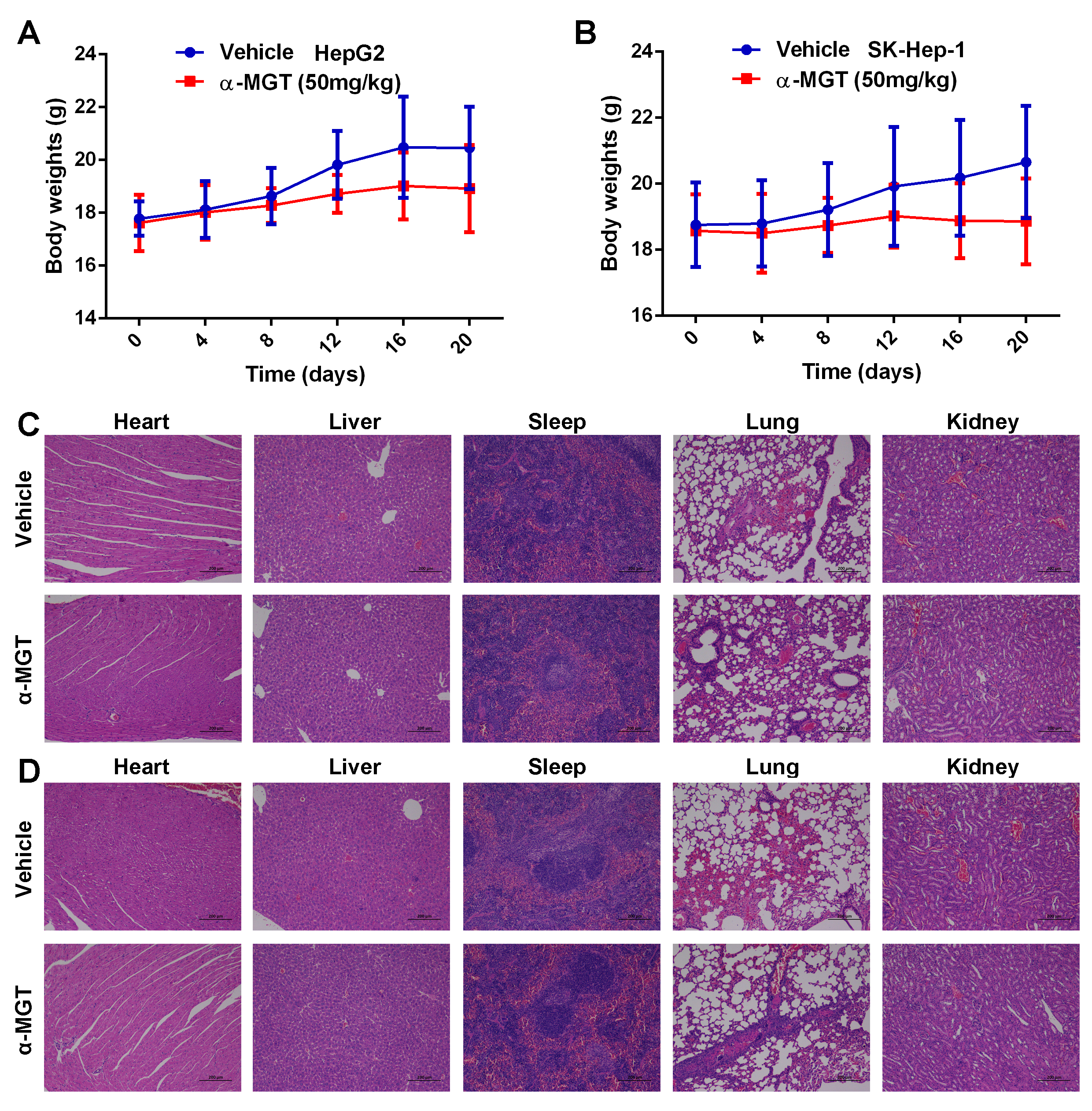

Supplement: Supplementary file 3 — Supplementary Figure 3 [file 41419_2020_2227_MOESM3_ESM.tif]

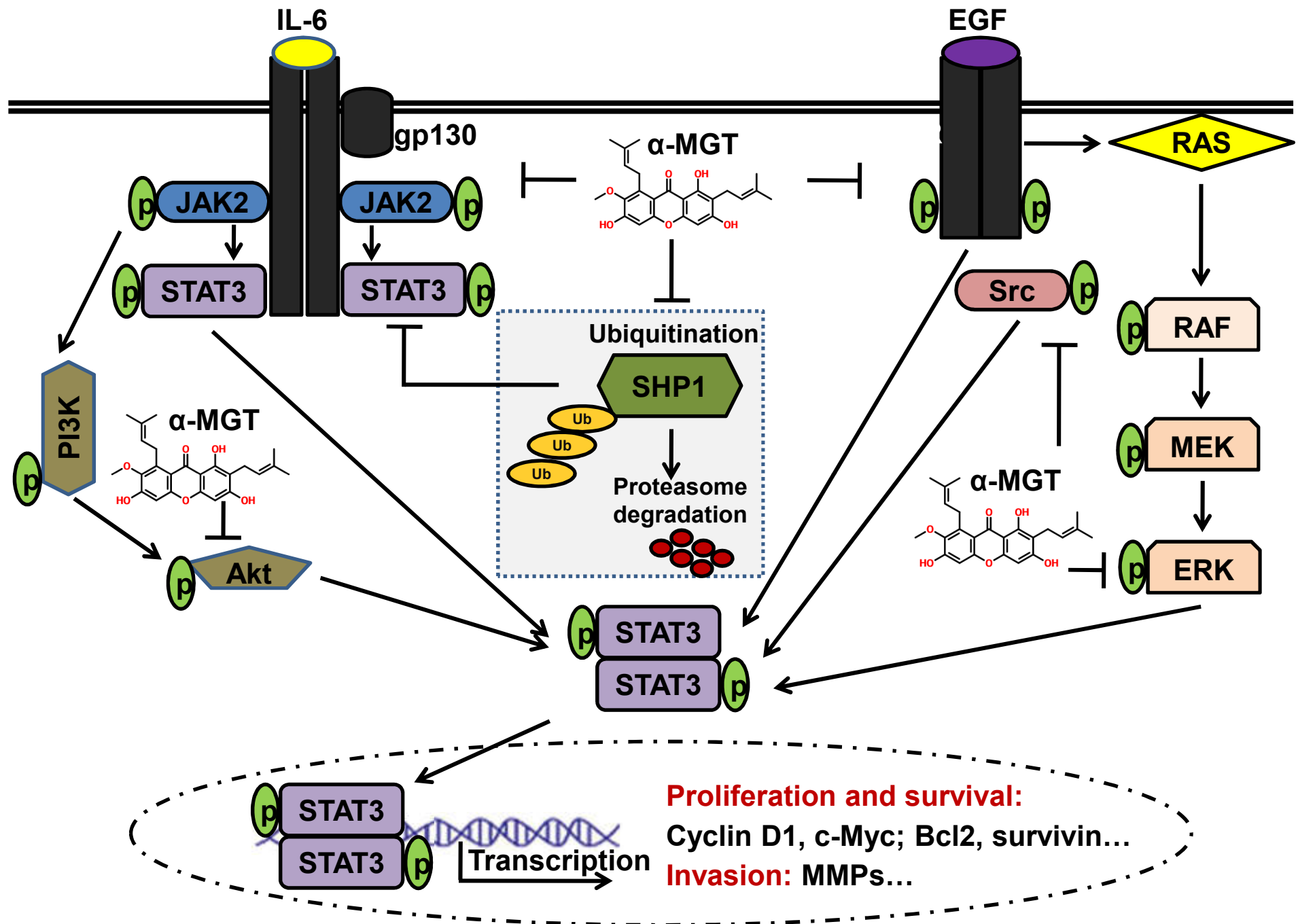

Supplement: Supplementary file 4 — Supplementary Figure 4 [file 41419_2020_2227_MOESM4_ESM.pdf]
